# Supplementary material for: A survey of early-career researchers in Australia
Source: eLife. 2021 Jan 11;10:e60613. doi: 10.7554/eLife.60613 (PMC7800379; doi:10.7554/eLife.60613)
Supplement: Supplementary file 2. [file elife-60613-supp2.docx]

**Supplementary file 2**

**Comparison of satisfaction data from this survey, and historical surveys in Australia.**

| **Study** | **Coates *et al*. 2009**  **(academics all stages)** | **Bexley *et al*. 2011**  **(ECRs)** | **Bell and Yates 2015**  **(STEM all stages)** | **NTEU 2017**  **(academics all stages)** | **Current study 2019 (ECRs)** |
| --- | --- | --- | --- | --- | --- |
| **Questions** | **Percent of respondents who agree** | | | | |
| I have good job security |  | 26% | 38% | 36% | 17% |
| My job is a source of considerable personal stress | 28% | 38% | 43% |  | 52% |
| This is not a good time for any young person to aspire to an academic career in my discipline | 36% | 39% | 46% |  | 65% |
| I have freedom to pursue my own research interests |  | 58% | 47% |  | 55% |
| Satisfaction with my job* | 55% | 62% | 71% | 78% | 62% |
| I feel my work is valued |  |  | 74% | 46% | 61% |

* For “Satisfaction with my job” questions, Bell & Bexley question: “Generally speaking I am satisfied with my job”. Other studies: “How would you rate your overall satisfaction with your current job”
